# Herbal Medicine “Shulifenxiao” Formula for Nephrotic Syndrome of Refractory Idiopathic Membranous Nephropathy

**DOI:** 10.3389/fphar.2021.675406

**Published:** 2021-05-10

**Authors:** Hailan Cui, Frank Qiang Fu, Baoli Liu, Wei Jing Liu, Yu Ning Liu

**Affiliations:** ^1^ Beijing Changping Hospital of Traditional Chinese Medicine, Beijing, China; ^2^ Renal Research Institution of Beijing University of Chinese Medicine, Key Laboratory of Chinese Internal Medicine of Ministry of Education and Beijing and Dongzhimen Hospital, Beijing University of Chinese Medicine, Beijing, China; ^3^ School of Chinese Materia Medica, Beijing University of Chinese Medicine, Beijing, China; ^4^ Beijing Hospital of Traditional Chinese Medicine Affiliated to Capital Medical University, Beijing, China; ^5^ Zhanjiang Key Laboratory of Prevention and Management of Chronic Kidney Disease, Guangdong Medical University, Zhanjiang, China

**Keywords:** Chinese herbal medicine, nephrotic syndrome, refractory membranous nephropathy, retrospective analysis, shulifenxiao formula

## Abstract

**Background:** Treatment for adult patients with refractory idiopathic membranous nephropathy (RIMN) by conventional immunosuppressive regimens is not satisfactory. This study aims to evaluate the effectiveness of Chinese herbal medicine, Shulifenxiao formula, as a promising regimen.

**Methods:** A total of 31 RIMN patients resistant to corticosteroid or immunosuppressive agents were retrospectively analyzed. Shulifenxiao treatment lasted a minimum of 12°months in all patients and extended to 24°months in 11 patients. The primary outcomes [the complete remission (CR) and partial remission (PR)] and secondary outcomes (the serum creatinine and estimated glomerular filtration rate (eGFR) levels) were measured at 6, 12, 18, and 24°months.

**Results:** The data provided an average follow-up of 21 ± 9.16°months from baseline. The remission was attained in 25/31 patients (80.7%: CR 29.0% and PR 51.6%) at 12°months and in 10/11 patients (90.9%: CR 54.6% and PR 36.4%) at 24°months, respectively. Proteinuria reduced from 6.02 g/d at baseline to 0.98 g/d at 12°months (*p* < 0.001) and to 0.27 g/d at 24°months (*p* = 0.003); serum albumin increased from 28 g/L to 37.2 g/L at 12°months (*p* < 0.001) and to 41.3 g/L at 24°months (*p* = 0.003); eGFR improved from 100.25 ml/min/1.73 m^2^ to 118.39 ml/min/1.73 m^2^ at 6°months (*p* < 0.001) and finally to 111.62 ml/min/1.73 m^2^at 24°months (*p* = 0.008). Only two patients developed subsequent relapse.

**Conclusion:** Shulifenxiao formula as a clinical cocktail therapy serves as an alternative therapeutic option for steroid and immunosuppressant-resistant RIMN patients, with a favourable safety profile, though further studies are warranted.

**Clinical Trial registration:** http://www.chictr.org.cn, Chinese Clinical Trials Registry [ChiCTR1800019351].

**GRAPHICAL ABSTRACT:**
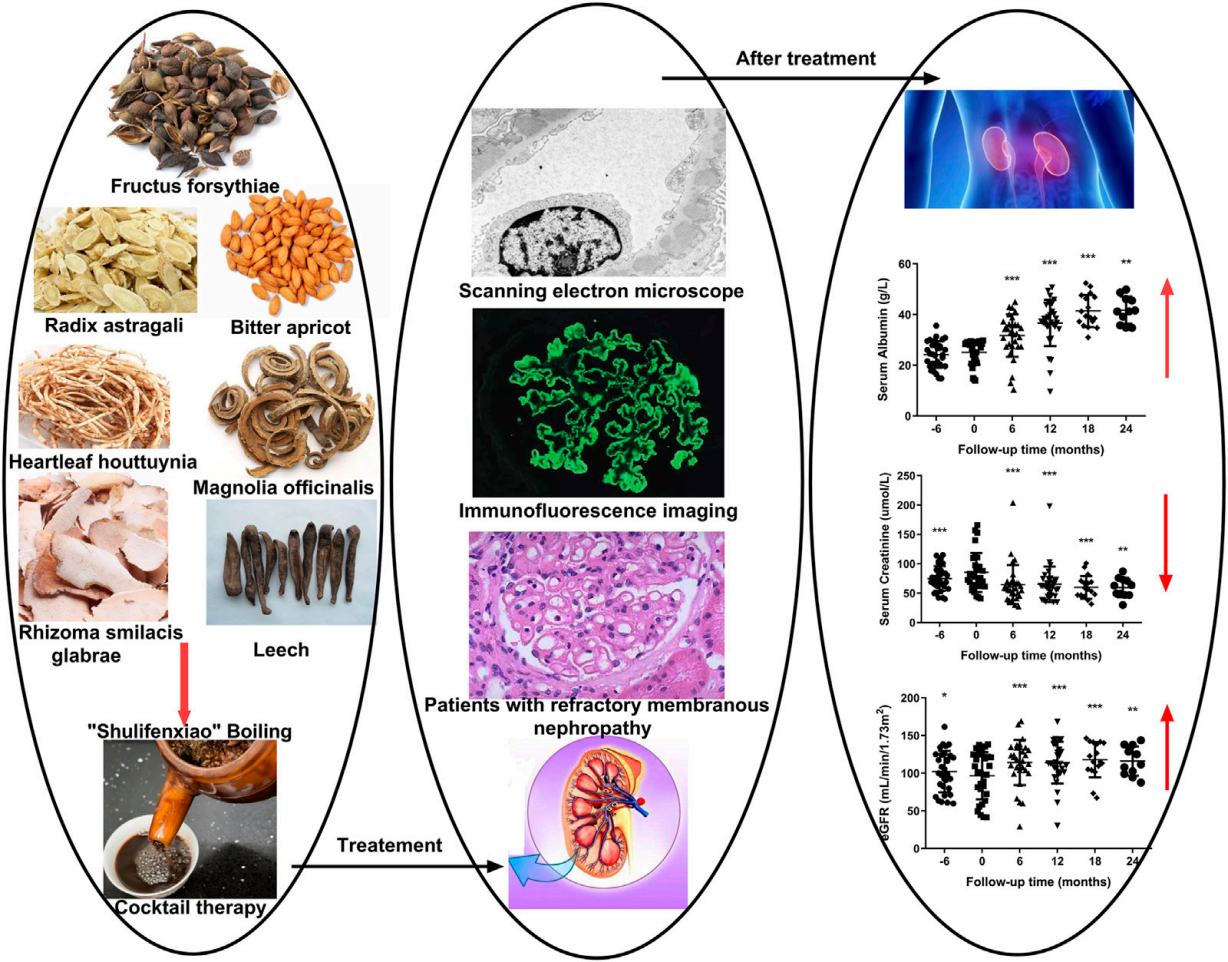


## Introdution

Membranous nephropathy (MN) is one of the most common causes of nephrotic syndrome in adults. Approximately 75% of MN cases are idiopathic (Glassock, 2010). Over the past few years, most MN patients have received glucocorticoid in combination with an alkylating agent, or received calcineurin inhibitor, according to the 2012 guidelines of kidney disease improving global outcomes (KDIGO). These regimens significantly improve the clinical remission rate and renal survival rate of idiopathic membranous nephropathy (IMN). Nevertheless, nearly 30% of patients fail to respond to the conventional immunosuppressive therapies and eventually develop end-stage renal disease (ESRD) or complications related to nephrotic syndrome (Du Buf-Vereijken et al., 2005; Couser, 2017). Therefore, refractory idiopathic MN (RIMN) is proposed to refer to MN cases resistant to steroids and general immunosuppressive agents (Iida et al., 2000; Chen et al., 2013; Sengupta et al., 2013). Treatment of RIMN is a significant challenge because traditional immunosuppressive agents appear to be unsuccessful and show obvious side effects. Alternative therapeutic agents to treat RIMN are an urgent need.

Based on the traditional theories of Traditional Chinese Medicine (TCM), TCM treatment has been done on humans for thousands of years with using the formula of natural herbal medicines. Up to now, TCM has accumulated rich experience in treating various kidney diseases and holds great potential for providing effective treatments for MN. Recently, existing preclinical and clinical evidence has supported the effectiveness of TCM in treating IMN. TCM treatment reduces proteinuria, improves serum albumin, and thereby prevents the progress of IMN and avoids side effects caused by long-term applications of immunosuppressive agents (Chen et al., 2013; Zhang et al., 2014; Xiao et al., 2018). Combination of TCM and Western medicine has proven to improve the outcome of MN.

Despite this, very few high-quality studies testing the efficacy of TCM in adult RIMN patients are available. This may be due to the difficulty in formulating a control group. So many of the current studies are small single arm studies or case reports, with the observation periods ranging from half a year to one year (Chen, 2016; Zhang et al., 2017). Therefore, for RIMN patients, the possible benefits of TCM remain undetermined.

Medicinal “Shulifenxiao” is an herbal treatment for qi-deficiency, a damp-heat syndrome of RIMN. It has been used for nearly 30 years as an empirical treatment, first devised by Professor Liu Yuning. We have found “Shulifenxiao” formula has a significant clinical effect on the treatment of MN, and so in 2014, we started the clinical data collect of MN patients receiving “Shulifenxiao” treatment to establish a database for MN analysis. Here, we conducted a retrospective study aimed to evaluate the efficacy, recurrence, and safety of the TCM Shulifenxiao formula in patients with RIMN who are resistant to conventional immunosuppressive regimens.

## Materials and Methods

### Patients

**FIGURE 1:**
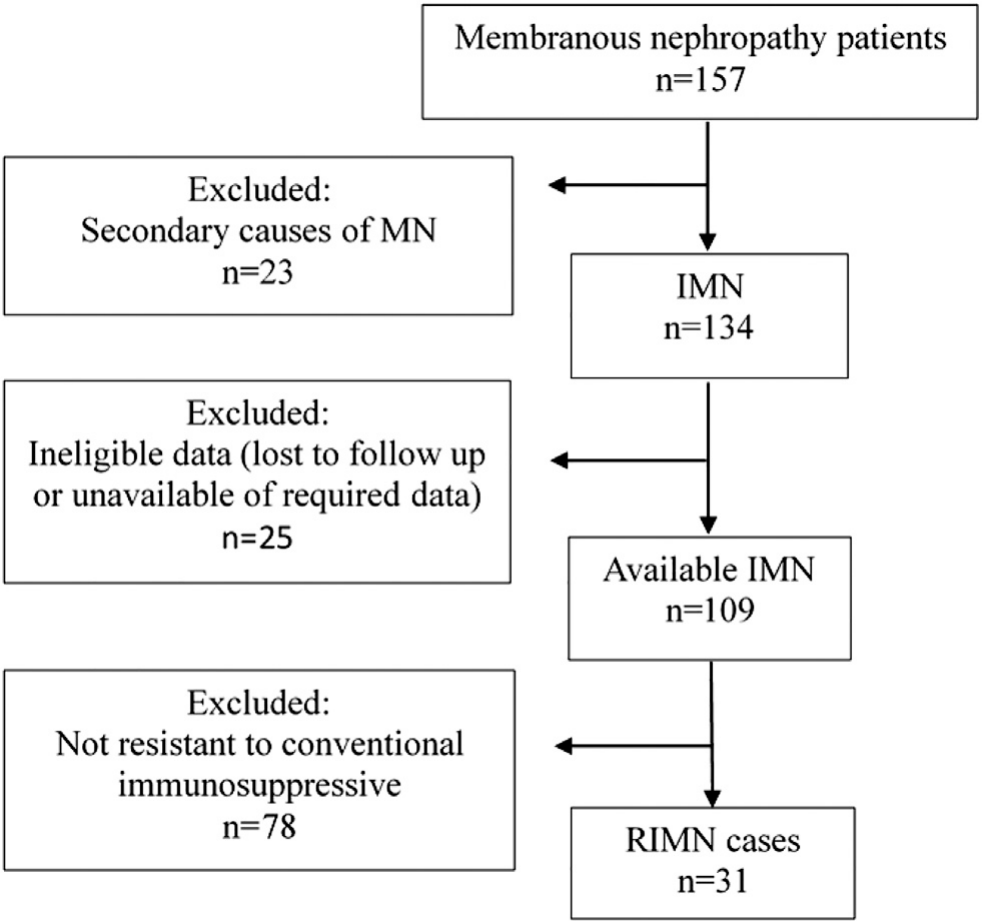
Flow chart of the patient selection process.

This was a retrospective case-series study. The outpatient records of adult patients with histologically proven membranous nephropathy in Dongzhimen Hospital affiliated to Beijing University of Chinese Medicine (Beijing, China) from January 2014 to June 2017 were screened (n = 157) (Figure 1). Only patients with the following criteria were included: 1) Histologically proven idiopathic membranous nephropathy; 2) Patients who did not respond to corticosteroid and immunosuppressive agents and remained in nephrotic syndrome (proteinuria≥3.5 g/d, serum albumin<30 g/L) even after 6°months of regular immunosuppressive therapy, such as prednisone (PRED), cyclophosphamide (CTX), cyclosporine A (CsA), tacrolimus (TAC), and mycophenolate mofetil (MMF); 3) eGFR > 30 ml/min/1.73 m^2^. 4) The follow-up period >1°year with an interval between visits of <3°months or >4°visit times per year.

31 patients were found to be eligible for the retrospective analysis.

### Intervention

All eligible patients were treated with a consecutive Shulifenxiao formula (a formula of TCM), which is made of seven Chinese herbs. Medicines was provided by the pharmacy department of Dongzhimen Hospital, and was orally administered 30 min after breakfast and supper (200°ml, bid). All patients were treated with angiotensin-converting enzyme inhibitors (ACEI) or angiotensin II receptor antagonist (ARB). If the patient's blood pressure remained >130/80 mmhg, appropriate calcium channel blockers were added to stabilize blood pressure. Treatment lasted for a minimum of 12°months in all patients. Clinical and laboratory data at 6°months prior to Shulifenxiao treatment, baseline, 6th, 12th, 18th, and 24th°months after Shulifenxiao treatment were analyzed. All adverse events recorded during follow-up were also analyzed.

### Outcome Measures

The primary outcomes were attainment of complete and partial remission and changes in proteinuria and serum albumin. Complete remission (CR) was defined as a reduction of proteinuria to <0.3 g/d and serum albumin >35 g/L; Partial remission (PR) was defined as level of proteinuria decline to <3.5 g/day but minimum >0.3 g/day and at least 50% reduction from baseline with a serum albumin concentration of at least 30 g/L; Nonremission (NR) was defined as a reduction of proteinuria <50% or proteinuria >3.5 g/d. Relapse was defined as an increased proteinuria >3.5 g/d in consecutive analyses in patients with CR or PR. Any relapse was considered as an endpoint of the study. If no relapse was observed prior to the latest visit, June 30, 2018, was designated as the endpoint. Secondary outcomes included changes in serum creatinine and eGFR levels at each time point.

### Statistical Analysis

Statistical analysis was performed with SPSS 20.0 (version 20.0; SPSS, Chicago, IL, United States). Values are given as mean ± standard deviation for normal distribution variables, and median with interquartile range for abnormal distribution continuous variables. The *t*-test was used for comparisons of the means of two normal distributed quantitative variables. One-way analysis of variance (ANOVA) was used for comparisons of the means of multiple normal distributed quantitative variables. For anomaly distributed qualitative variables, a paired nonparametric test was used. A *p* value < 0.05 was considered to be statistically significant.

## Results

### General Information

Characteristics of the 31 patients at baseline are listed in Table 1. All patients were treated with Shulifenxiao formula for a minimum of 12°months. Of these, 17 patients had complete clinical and laboratory data for 18°months of treatment, and 11 patients had complete data of 24°months of treatment. The average follow-up time was 21 ± 9.16°months. Other diseases associated with MN include 11 patients with hypertension, 1 with diabetes, 6 with hyperlipidemia, 1 with hyperuricemia, and 2 with coronary heart disease. The previous immunosuppressive agents used in the 31 patients were shown in Table 2.

**TABLE 1:** Characteristics of patients at baseline.

Characteristics	Baseline (*n* = 31)
Gender [*n* (male/female)]	23/8
Age (years)	44 (28–53)
Duration of disease (months)	12 (8–36)
Systolic BP (mmHg)	128.77 ± 11.88
Diastolic BP (mmHg)	77.9 ± 9.1
Histology grading of membranous nephropathy (*n* [%]) stage I	13 (41.9)
Stage II	10 (32.3)
Stage III	2 (6.5)
Non-typical membranous nephropathy	6 (19.3)
Proteinuria (g/d)	6.02 (5.13–8.8)

Proteinuria Group [*n* (%)]	
4–8 g/d	22 (71)
>8 g/d	9 (29)
Serum Albumin (g/L)	28 (21.8–28.9)
Serum Creatinine (umol/L)	78 (61.9–97.2)
eGFR (mL/min/1.73 m^2^)	100.24 (72.73–124.7)

eGFR Group [*n* (%)]	
>90 ml/min/1.73 m^2^	18 (58.0)
60–90 ml/min/1.73 m^2^	7 (22.6)
<60 ml/min/1.73 m^2^	6 (19.4)
ACEI [*n* (%)]	20 (64.5)
ARB [*n* (%)]	11 (35.5)

Results are presented as mean ± standard deviation, median with inter-quartile range, or number of patients (percentage).

**TABLE 2:** The previous immunosuppressive agents used in patients.

Previous treatment regimen	Samples	Percentage (%)
PRED, CTX	6	19.35
CsA	3	9.68
CsA, PRED	6	19.35
CsA, TAC	1	3.23
CsA, PRED, TAC	1	3.23
TAC	4	12.90
PRED, TAC	3	9.68
PRED, CTX, CsA	3	9.68
PRED, CTX, TAC	1	3.23
PRED, TAC, MMF	2	6.45
PRED, CsA, MMF	1	3.23

CsA, cyclosporine A; CTX, cyclophosphamide; MMF, mycophenolate mofetil; PRED, prednisone; TAC, tacrolimus.

### Primary Outcomes

**FIGURE 2:**
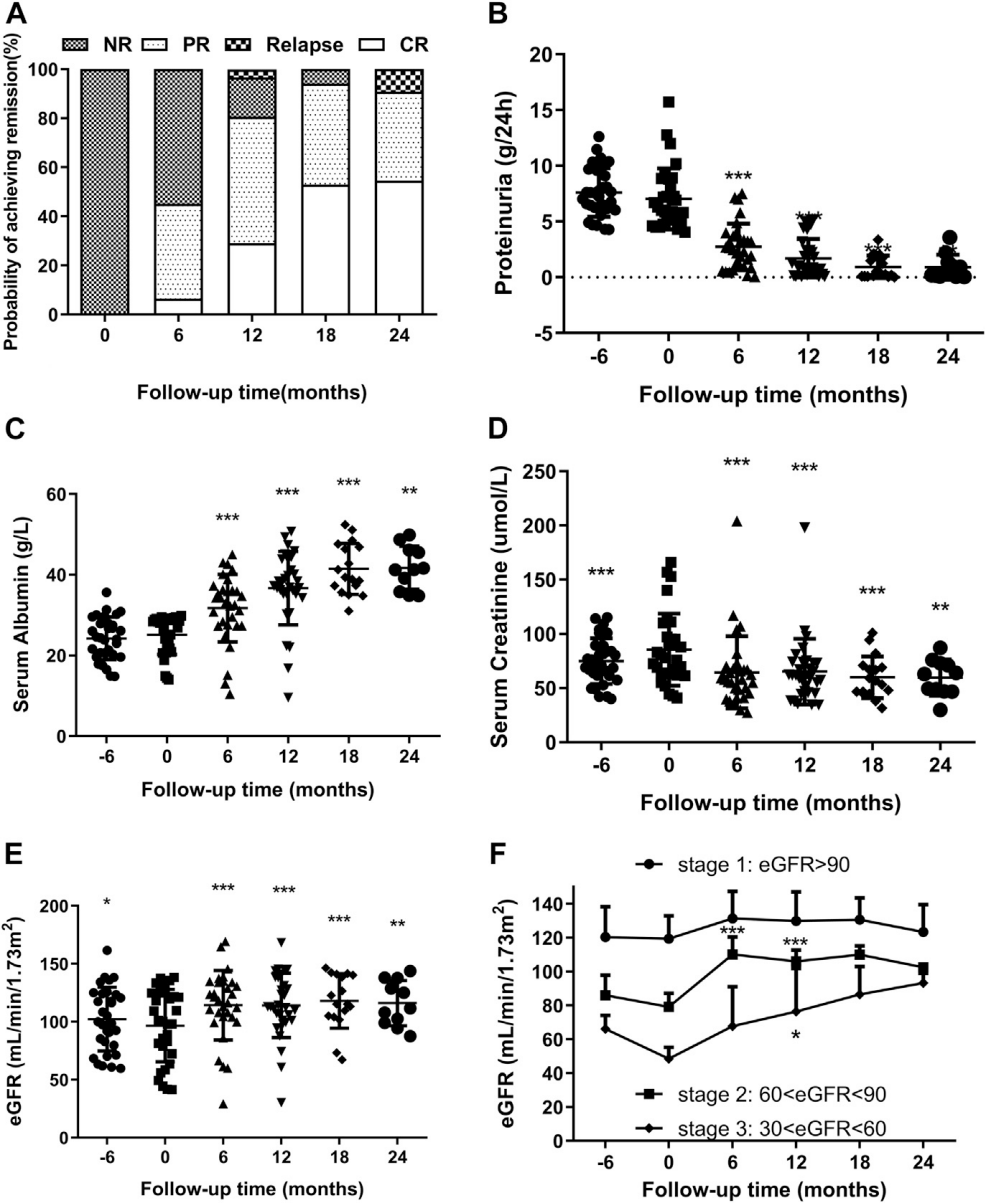
**(A)**. Remission and relapse rates of RIMN paitients with follow-up time (in months after starting Shulifenxiao formula therapy). **(B,C)** Changes of proteinuria and serum albumin levels (median with inter-quartile ranges) with follow-up time in RIMN patients (−6°months means 6°months before Shulifenxiao therapy). The values of each time point were compared to the baseline (0°months), and the *p* values were evaluated by paired non-parametric test. *p values < 0.05, **p values < 0.01, ***p values < 0.001. **(D,E)** The changes of serum creatinine and eGFR levels (median with interquartile ranges) with follow-up time in RIMN patients. **(F)**. The changes of eGFR levels (mean ± standard deviation) in RIMN patients of three stages with follow-up time (−6°months means 6°months before Shulifenxiao therapy). The values of each time point were compared to one’s own baseline. The *p* values of **(D,E)** were evaluated by a paired non-parametric test, and the *p* values of **(F)** were evaluated by one-way ANOVA test.*p values < 0.05, **p values < 0.01, ***p values < 0.001.

The partial and complete remission rates increased gradually with the extension of treatment time (Figure 2A). The remission rate was 45.2% (14/31; CR 6.5% and PR 38.7%) at 6°months, 80.7% at 12°months (25/31; CR 29.0% and PR 51.6%), and 90.9% at 24°months (10/11; CR 54.6% and PR 36.4%). The average length from treatment initiation to PR was 7.32 ± 3.61°months, while to CR was 12.38 ± 4.73°months. Notably, two (6.45%) patients presented relapse within 24°months. One patient who achieved CR at 6°months relapsed at 12°months (due to a urinary tract infection). Another PR patient relapsed at 24°months (due to an upper respiratory infection), which indicated the end of the follow-up.

The changes in proteinuria and serum albumin levels in RIMN patients are illustrated in Figures 2B,C. The proteinuria levels decreased drastically from 6.02 (5.13–8.80)°g/d at baseline to 0.98 (0.27–2.41)°g/d at 12°months (*p* < 0.001) and further to 0.27 (0.1–1.34)°g/d at 24°months (*p* = 0.003). Meanwhile, serum albumin gradually increased from 28 (21.8–28.9)°g/L at baseline to 37.2 (35.2–43.7)°g/L at 12°months (*p* < 0.001) and to 41.3 (35.8–46.1)°g/L at 24°months (*p* = 0.003). Levels of both test indices showed a significant difference between baseline and different post-treatment time points.

It is well known that MN has a high spontaneous remission rate (Saito et al., 2017), and it can be concluded that some patients achieve spontaneous remission. In order to exclude such potential spontaneous remission cases, we reviewed the proteinuria and serum albumin levels 6°months prior to the TCM treatment. Data showed that there were no significant changes in these levels in the prior 6°months (*p* > 0.05).

### Secondary Outcomes

Another important finding of this study was the reduction in serum creatinine. Although the level of serum creatinine increased from 6°months prior to TCM treatment (*p* < 0.001), it decreased remarkably from 78 (61.997.2)°g/L at baseline to 58 (45.23–68)°g/L at 6°months (*p* < 0.001) and continued to fluctuate smoothly until reaching 63 (47–73)°g/L at 24°months (*p* = 0.003) (Figure 2D). Simulaneously, eGFR significantly increased from 100.25 (72.37–124.70) at baseline to 118.39 (104.09–133.86)°ml/min/1.73 m^2^ at 6°months (*p* < 0.001) and finally to 111.62 (99.01–137.83)°ml/min/1.73 m^2^ at 24°months (*p* = 0.008) (Figure 2E).

Next, we observed changes in eGFR values. To the best of our knowledge, according to the KDOQI clinical guidelines, chronic kidney disease (CKD) can be classified into different stages (Levey 2003): Stage 1: kidney damage with normal or increased GFR (≥90 ml/min/1.73 m)^2^; Stage 2: kidney damage with mild decreased GFR (60–89 ml/min/1.73 m)^2^; Stage 3: moderately decreased GFR (30–59 ml/min/1.73 m)^2^; Stage 4: severely decreased GFR (15–29 ml/min/1.73 m)^2^; Stage 5: kidney failure with GFR <15 ml/min/1.73 m^2^ or dialysis required. As shown in Figure 2F, although there was no significant difference from baseline (*p* > 0.05), eGFR values in stage 1 were still on the rose after treatment. Interestingly, the eGFR values of the stage 2 group were statistically enhanced from 79.14 ± 8.63 ml/min/1.73 m^2^ at baseline to 110.16 ± 11.14 ml/min/1.73 m^2^ at 6°months, and reached 106.02 ± 7.22 ml/min/1.73 m^2^ at 12°months, and a significant difference was observed compared to baseline (*p* < 0.001). A similar trend was observed in the group of CKD stage 3: the eGFR values increased from 48.58 ± 7.34 ml/min/1.73 m^2^ at baseline to 67.85 ± 25.27 ml/min/1.73 m^2^ at 6°months (*p* = 0.167), and to 76.39 ± 27.92 ml/min/1.73 m^2^ at 12 months (*p* = 0.04). Since there were fewer than 5 cases in stages 2 and 3 during the 18th and 24th°month, we did not compare them. From the above results, it can be seen that Shulifenxiao formula treatment can improve the glomerular filtration rate, especially in patients with low eGFR.

### Safety

Over the follow-up time of 31 patients, liver function, routine blood tests, and blood pressure in all the patients indicated no significant changes from baseline after the intervention (Table 3). No severe adverse events were reported. Only one patient developed nausea, and another patient developed vomiting, but both were transient and the symptoms resolved after 2 weeks.

**TABLE 3:** Side effects of patients at baseline and the last follow-up of the study.

Characteristics	Baseline	Last follow-up	*p*
ALT (U/L)	19.11 ± 8.20	18.47 ± 6.21	0.536
AST (U/L)	18.95 ± 8.75	18.04 ± 5.36	0.603
Total bilirubin (umol/L)	8.68 ± 2.92	8.87 ± 3.29	0.799
White blood cell (×109/L)	7.48 ± 2.45	7.26 ± 1.29	0.608
Hemoglobin (g/L)	132.87 ± 15.22	133.61 ± 12.83	0.574
Platelet count (×10^9^/L)	243.39 ± 64.32	243.45 ± 55.74	0.989
SBP (mm Hg)	128.77 ± 11.88	127.13 ± 8.90	0.17
DBP (mm Hg)	77.90 ± 9.10	76.94 ± 7.66	0.22

ALT, alanine aminotransferase; AST, aspartate transaminase; DBP; diastolic blood pressure; SBP, systolic blood pressure.

## Discussion

The treatment for adult patients with RIMN who are resistant to standard immunosuppressive therapies remains a therapeutic challenge. The efficacy and safety profile of conventional treatments like steroids, CTX, CsA, TAC is not always satisfactory as revealed by recent studies. W. Chen et al. (Chen et al., 2013) reported a benefit of combining TAC with prednisone in treating 14 adults Chinese RIMN patients, that remission was attained in 11/14 patients (78.6%: CR 35.7% and PR 42.9%) at the end of follow-up. T. Saito et al. (Saito et al., 2017) treated 19 patients with Mizoribine (MZR) in combination with prednisolone, and CR was attained in only 10/19 (52.6%) patients after more than 2°years of treatment. F.B. Cortazar et al. (Cortazar et al., 2017) reported a higher remission rate in a study combining low-dose rituximab with oral cyclophosphamide, and an accelerated prednisone taper in treating 15 consecutive IMN patients (including 8 RIMN patients), in which 100% of patients achieved PR and 93% of patients achieved CR at a median time of 2 and 13°months respectively. Regardless of their fair or good efficacy, these therapies have serious adverse events, sometimes with fatal consequences. Therefore, in China, many RIMN patients turn to traditional Chinese medicine (TCM) for alternative therapy. In this study, we retrospectively reviewed the efficacy and safety of Shulifenxiao formula in 31 adult RIMN patients who did not response to 6°months conventional immunosuppressant therapies.

In this study, we excluded cases with spontaneous remission potentials by confirming no changes in the proteinuria, serum albumin levels of all patients from 6°months prior TCM treatment to the baseline. After Shulifenxiao treatment, a significant decrease in proteinuria from 6.02 g/d at baseline to 0.27 g/d at 24°months and an upward trend in serum albumin level was achieved. Previous studies have suggested that patients with proteinuria of 0.3–1.0 g/d showed a favourable prognosis almost equal to CR (Shiiki et al., 2004; Saito et al., 2014), and patients with PR had a similar average GFR decline as those with CR (Troyanov et al., 2004). From this viewpoint, we can consider that most participants of this study achieved remission within 2°years of treatment, which is cross verified with the results in the remission rate. Obviously, Shulifenxiao formula has shown a promising effect in inducing remission in the refractory patients and relatively low relapse rate.

Persistence of nephrotic syndrome in MN portends a poor prognosis, with a considerably high risk of a GFR decline rate of 10 ml/min/1.73 m^2^, and a 29% ESRD risk rate (Troyanov et al., 2004), therefore long-term immunosuppressive therapies are used. However, these therapies do not always induce remission and may cause significant adverse effects. The calcineurin inhibitor (CsA and TAC) directly affects renal function and cause nephrotoxicity and hypertension. Therefore, under these treatments, improvement of proteinuria and protection of renal function seems to be a trade-off process. Notably, Shulifenxiao formula appeared to show significant benefits in increasing eGFR levels, especially in CKD stage 2: The eGFR values increased from 79.14 to 110.16 ml/min/1.73 m^2^ at 6°months (*p* < 0.001). These findings suggest that Shulifenxiao formula can improve renal function by significantly increasing eGFR, which is important for long-term prognosis.

In the present study, no severe adverse events were observed. Liver function, routine blood tests and blood pressure in all patients indicated no significant changes between baseline and post-treatment time points. Only two patients developed nausea or vomiting, both of which were mild and easily controlled. This is clearly an exciting outcome for RIMN patients who have to take long-term immunosuppressants, most of which are associated with serious adverse events. Compared to these conventional drugs, Shulifenxiao formula seems to be a safe and beneficial solution for long-term use.

**FIGURE 3:**
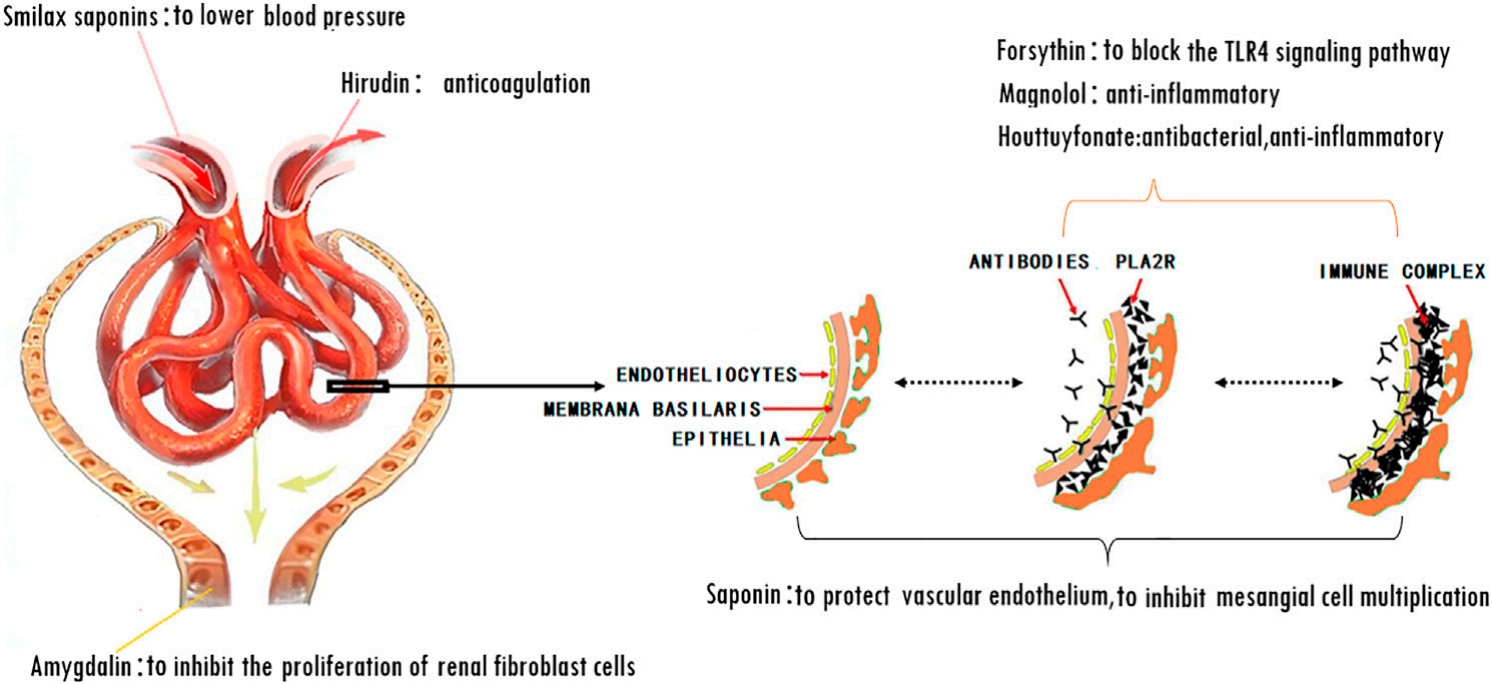
Shulifenxiao formula as clinical cocktail therapy has the potential to reduce proteinuria and attenuate kidney injury through these mechanisms.

The mechanism of action for herbal medicines have been studied, and their effects are mainly related to anti-inflammation, antioxidation, antifibrosis, immune system regulation, anticoagulation, and improvement of metabolic disturbance (Zhang et al., 2014; Ren et al., 2017; Wojcikowski et al., 2006). There are 7 Chinese herbal medicines in the Shulifenxiao formula (Table 4). One such component is Saponin in *Astragalus* which can improve immunity, protect vascular endothelium, inhibit mesangial cell multiplication, encourage the metabolism of body liquid, improve hemorheological targets, protect podocytes, and reduce renal interstitial fibrosis and glomerular sclerosis (Wang et al., 2017). Forsythin in Forsythia can effectively block the TLR4 signaling pathway and further by blocking the expression of inflammatory factors induced by lipopolysaccharide (Toba et al., 1991). Amygdalin in Bitter Apricot can increase the activity of type I collagenase in renal fibroblast cells in order to reduce the expression of type I collagen protein, that would further inhibit the proliferation of human renal fibroblast cells or would promote renal fibroblast apoptosis (Zhao and el al., 2020). Houttuyfonate in Houttuynia cordata has effective antibacterial, antiviral, enhance the body's immunity, diuresis (Good for peeing), and lower blood pressure (Wang et al., 2010). Smilax saponins in Magnolia Officinalis has the Anti-inflammatory, analgesic, antibacterial, antioxidant (Wang, 2014). Hirudin in Leech has the anticoagulation, anti-thrombosis, to reduce the deposition of fibrin-related antigen in the glomerulus in order to reduce the proliferation of mesangial cells and glomerular sclerosis (Ren et al., 2019) (Table 4). Cocktail therapy contains a combination of different medicines, taken by HIV/AIDS patients to improve health. Chinese officials currently administer cocktail therapy for treatment of HIV and COVID-19 (Schimt et al., 1996). The superior antiproteinuric and renoprotective effects of Shulifenxiao formula on RIMN may be related to multifactorial effects. These studies suggest that the Shulifenxiao formula as clinical cocktail therapy has the potential to reduce proteinuria and attenuate kidney injury through these mechanisms (Figure 3).

**TABLE 4:** Medicinal ingredients of Shulifenxiao formula.

Crude drug name	Part used	(g)	Main chemicals	Chemical structure	Pharmacological activity	Clinical impacts to this kidney disease
Radix Astragali	Root	60	Saponin	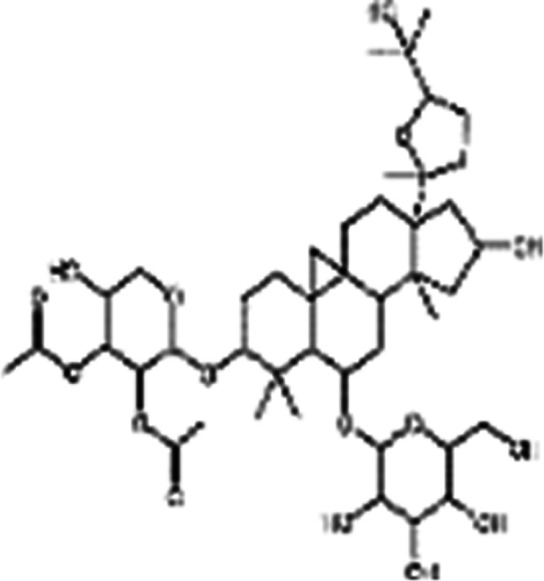	To improve immunity	Improve immunity, protect vascular endothelium, inhibit mesangial cell multiplication, encourage the metabolism of body liquid, improve hemorheological targets, protect podocytes, and reduce renal interstitial fibrosis and glomerular sclerosis
Fructus forsythiae	Fruit	15	Forsythin	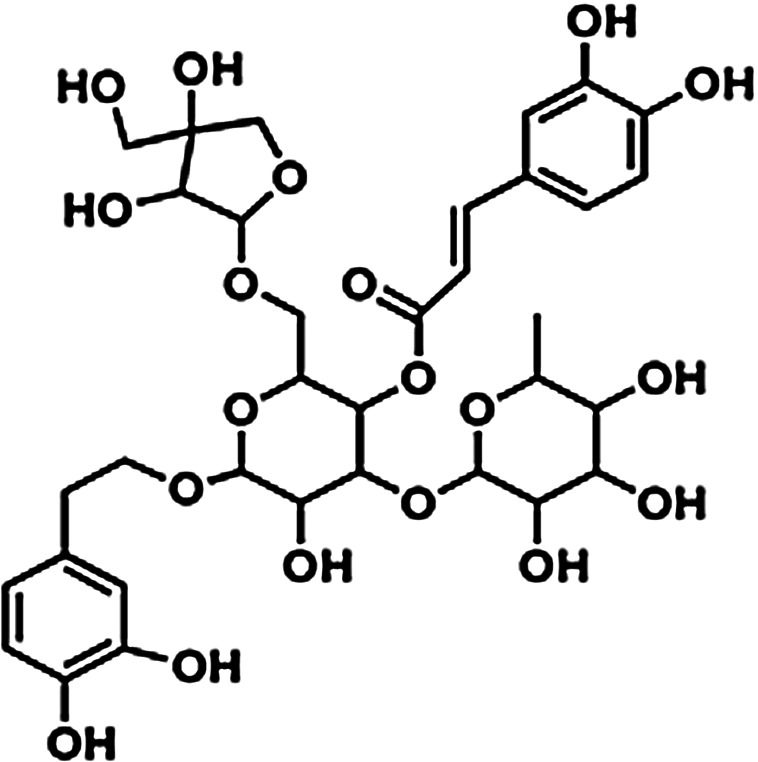	Antibacterial anti-inflammatory	It can effectively block the TLR4 signaling pathway and further by blocking the expression of inflammatory factors induced by lipopolysaccharide
Bitter Apricot	Seed	12	Amygdalin	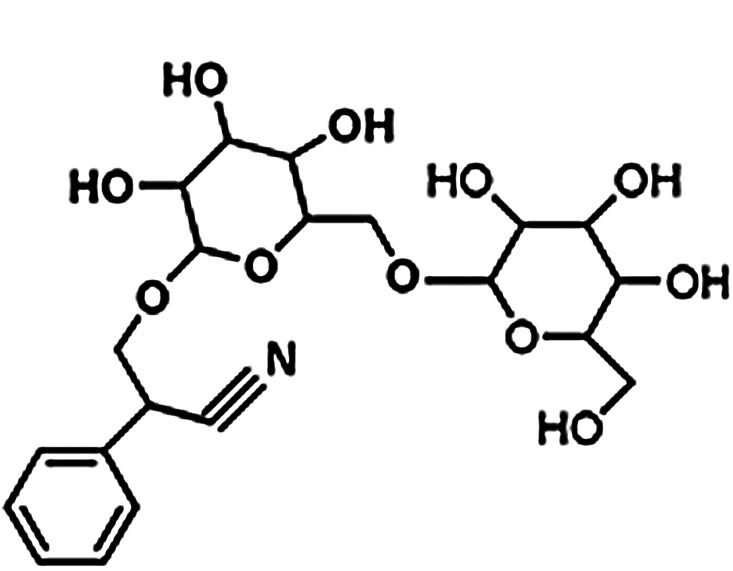	Expectorant and anti-inflammatory	To increase the activity of type I collagenase in renal fibroblast cells in order to reduce the expression of type I collagen protein, that would further inhibit the proliferation of human renal fibroblast cells or would promote renal fibroblast apoptosis
Heartleaf Houttuynia	Whole	30	Houttuyfonate	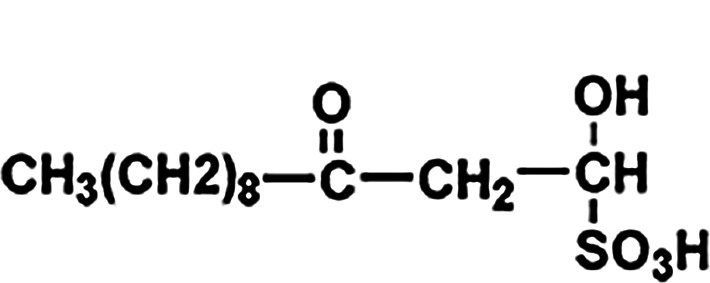	Antibacterial anti-inflammatory	Effective antibacterial, antiviral, enhance the body’s immunity, diuresis (Good for peeing), and lower blood pressure
Magnolia Officinalis	Bark	15	Smilax saponins	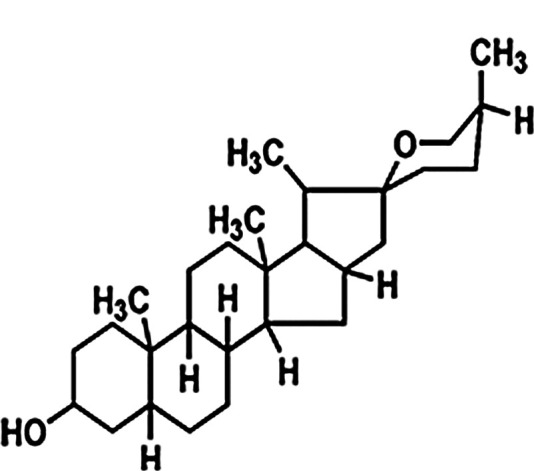	To improve immunity and anti-inflammatory	It has a muscle relaxation effect; it lowers blood pressure, anti-pathogenic microorganisms, anti-tumor; also, anti-platelet
Rhizoma smilacis glabrae	Root	30	Magnolol	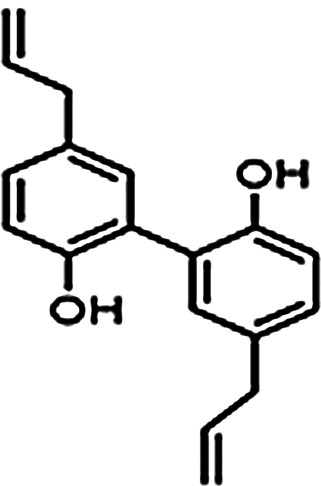	Antibacterial	Anti-inflammatory, analgesic, antibacterial, antioxidant
Leech	Whole	9	Hirudin	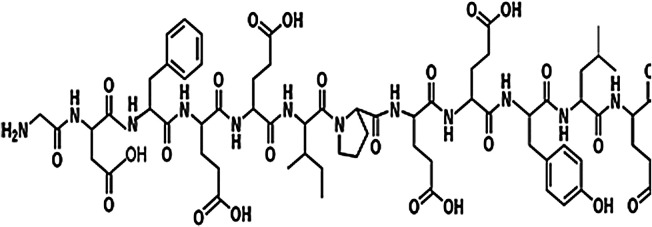	Anticoagulant	Anticoagulation, anti-thrombosis, to reduce the deposition of fibrin-related antigen in the glomerulus in order to reduce the proliferation of mesangial cells and glomerular sclerosis

Our study has the advantages and innovation as this is the first report or information about “Herbal Medicine “Shulifenxiao” therapy for Nephrotic Syndrome of Refractory Idiopathic Membranous Nephropathy” in the world. Most of the previous clinical studies of TCM were the research of IMN and they compared the differences of the efficacy and safety between TCM and traditional immunosuppressant. There were few studies of RIMN. Our study focusses on RIMN, and we strictly selected the patients with nephrotic syndrome after 6 months of treatment with a standardized immunosuppressant regimen.

The major limitation of this study is that it is a retrospective single-centre study, with a limited number of patients, and lacking a parallel control group. As a matter of fact, In China, before most MN patients came to our hospital for the herbal medicine therapy, patients suffering from MN have previously received conventional treatment such as immunosuppressant or hormone therapy. Some patients cannot sustain the side effects of immunologic therapy or ineffective treatment options, and so they prefer to accept to the herbal medicine treatment. Based on real-world conditions and patients’ preference for herbal treatment, all patients were treated with traditional herbal medicine, with no patients treated with immunosuppressant or hormones therapy only. This made it difficult to formulate a control group. Therefore, the statistical results must be interpreted with caution in this setting. Meanwhile, randomized controlled trials with long-term follow-up are needed in the future to establish Shulifenxiao formula as an evidence-based treatment option for RIMN patients.

## Conclusion

In this study, patients with refractory membranous nephropathy were treated with Shulifenxiao cocktail therapy. After treatment, it was observed that patient urine protein content decreased, plasma albumin levels increased, clinical remission rate was significantly increased, and patient's renal function was improved.

Shulifenxiao formula as clinical cocktail therapy serves as an alternative therapeutic option for steroid and general immunosuppressant-resistant RIMN patients, with a favourable safety profile, though studies with larger sample sizes and longer follow-ups are warranted. When seeking new RIMN treatment, we believe herbal medicine therapy should be considered.

## Data Availability

The raw data supporting the conclusion of this article will be made available by the authors, without undue reservation.

## Abbreviations

ACEI, angiotensin converting enzyme inhibitors; ALT, alanine aminotransferase; ANOVA, one-way analysis of variance; ARB, angiotensin II receptor antagonist; AST, aspartate transaminase; CKD, chronic kidney disease; CR, complete remission; CsA, cyclosporine A; CTX, cyclophosphamide; DBP, diastolic blood pressure; eGFR, estimated glomerular filtration rate; ESRD, end-stage renal disease; IMN, idiopathic membranous nephropathy; KDIGO, kidney disease improving global outcomes; MMF, mycophenolate mofetil; MN, membranous nephropathy; MZR, mizoribine; NR, nonremission; PR, partial remission; PRED, prednisone; RIMN, refractory idiopathic membranous nephropathy; SBP, systolic blood pressure; TAC, tacrolimus; TCM, traditional Chinese medicine.
